# Screening of prognosis-related Immune cells and prognostic predictors in Colorectal Cancer Patients

**DOI:** 10.1186/s12885-023-10667-y

**Published:** 2023-03-01

**Authors:** Shuangshuang Deng, Qiping Zhu, Hongyan Chen, Tianyu Xiao, Yinshen Zhu, Jinli Gao, Qing Li, Yong Gao

**Affiliations:** 1 Department of Pathology, Shanghai East Hospital, School of Medicine, Tongji University, Shanghai, 200092 China; 2 Department of Neurology, Luodian Hospital, Baoshan District, Shanghai, 201908 China; 3 Department of Oncology, Shanghai East Hospital, School of Medicine, Tongji University, Shanghai, 200092 China

**Keywords:** Colorectal cancer, Immune cells, Prognosis, CD163, CXCR5, CD4

## Abstract

**Objective:**

To accurately screen potential immune cells that can predict the survival of colorectal cancer (CRC) patients and identify related prognostic predictors.

**Methods:**

The sample data of CRC patients were downloaded from the GEO database as a training set to establish a prognosis-scoring model and screen prognosis-related immune cells. The sample data of CRC patients from the TCGA database were used as the validation set. Simultaneously, cancer tissue samples from 116 patients with CRC diagnosed pathologically in Shanghai Dongfang Hospital were collected to analyze the relationship of prognosis-related immune cells with patients’ survival, and clinical and pathological parameters, and to screen prognostic predictors.

**Results:**

Prognosis-related immune cells screened from GEO and TCGA databases mainly included Follicular Helper T cells (Tfh), Monocytes and M2 Macrophages. In the training set, the 2,000- and 4,000-day survival rates were 48.3% and 10.7% in the low-risk group (N = 234), and 42.1% and 7.5% in the high-risk group (N = 214), respectively. In the validation set, the 2,000- and 4,000-day survival rates were 34.8% and 8.6% in the low-risk group (N = 187), and 28.9% and 6.1% in the high-risk group (N = 246), respectively. The prognosis of patients in the high-risk group was worse than that in the low-risk group (P < 0.05). Furthermore, the screened primary prognostic predictors were CD163 and CD4 + CXCR5. CD163 protein expression was distributed in Monocytes and M2 Macrophages. The 1,000- and 2,000-day survival rates were 56.1% and 7.0% in the CD163 low-expression group, and 40.7% and 1.7% in the high-expression group (N = 214), respectively, showing a worse prognosis in the high-expression group than that in the low-expression group. Meanwhile, the immune marker CD4 + CXCR5 could identify Tfh. The 1,000- and 2,000-day survival rates were 63.9% and 5.6% in the CD4 + CXCR5 high-expression group, and 33.3% and 2.8% in the low-expression group (N = 214), respectively, with a better prognosis in the high-expression group than that in the low-expression group.

**Conclusion:**

Prognostic-related immune cells of CRC mainly include Tfh cells, Monocytes and M2 Macrophages. Monocytes and M2 Macrophages correlate negatively, while Tfh cells correlate positively with the prognosis of CRC patients. Immune markers CD163 and CD4 + CXCR5 can be considered as the prognostic predictors of CRC with clinical value of the application.

Colorectal cancer (CRC) is one of the most common malignant tumors in the world, which is the third most common cancer and the second leading cause of cancer deaths worldwide [1]. According to statistics, the number of newly diagnosed CRC cases in China was 521,590 in 2018, accounting for 12.2% of all new cancer cases; and the number of CRC-related deaths was 247,563, accounting for 8.6% of all cancer fatalities [2]. Meanwhile, as evidenced by the latest statistics, there were 104,270 newly diagnosed CRC cases and 52,980 cancer deaths in the United States by 2021 [3]. Patients with early CRC have a good prognosis, with a 5-year survival rate of 82-91%. However, the majority of patients were in the advanced stage at diagnosis clinically, with a 5-year survival rate as low as 12% [4].

Considering the high heterogeneity and complexity of CRC, there may be different prognostic outcomes of patients at different stages, highlighting the requirement for doctors to reasonably assess the risk of CRC, so as to facilitate subsequent individualized treatment [5, 6]. At present, the Tumor Node Metastasis(TNM) stage has been recognized to be the most common option for prognosis monitoring, which, however, is only of reference significance for CRC patients at the early stage. Moreover, the prognosis of CRC patients is a multifactorial process that may be related to genetics, epigenetic status and tumor microenvironment (TME) of CRC [7]. It has been reported with regard to the prognostic value of some immune-related components in TME. For instance, higher levels of Th1 cell infiltration in CRC are associated with better disease-free survival, while Th17 cell infiltration may indicate poor prognosis [8]. Therefore, further identification of immune cells and related prognostic predictors have broad prospects in predicting the survival of CRC patients clinically. It can complement the traditional method of the TNM stage to enhance the predictive power for the prognosis of CRC patients.

## Materials and methods

### Screening of prognosis-related immune cells based on GEO and TCGA databases

#### Data source

From July 25 to August 31, 2021, three independent transcriptional datasets were selected, including TCGA-COAD (N = 457), GSE39582 (N = 585) and GSE41258 (N = 182). Exclusion criteria: cases without follow-up data or clinical data. GSE39582 and GSE41258 datasets were used as the training sets and TCGA datasets as the validation set. The clinical data and gene expression matrix of this study were downloaded from TCGA (https://portal.gdc.cancer.gov) or GEO (https://www.ncbi.nlm.nih.gov/geo).

#### Acquisition of differential immune cells in CRC

CIBERSORT deconvolution algorithm was employed to analyze immune cell infiltration via simulation calculation (100 times) of the characteristic matrix of transcriptional datasets involving 22 types of immune cells such as T cells, B cells and NK cells. Data with P < 0.05 were incorporated for subsequent analysis.

1.1.3 Construction and evaluation of immune-elated prognostic risk model.

With the exclusion of CRC patients with clinical data that were not registered in the GEO database and inaccurate follow-up data of survival, univariate analysis was conducted to screen immune cells that had a significant association with the prognosis of CRC (P < 0.05). In order to avoid the risk of data over-fitting, the ‘glmnet’ package in R software was used to apply Lasso Cox regression to select immune cells with significant prognostic value as the molecules for modeling. Finally, multivariate Cox regression was adopted to identify independent prognosis-related immune cells. At the same time, the risk value formula was established based on the weight coefficient and gene expression calculated by Cox regression. The constructed formula was then utilized to calculate the prognostic risk score for each patient. Patients were further divided into the high-risk group and low-risk group according to the cut-off value of the median risk score. The relationship between prognosis-related immune cells and the overall survival (OS) of patients was analyzed by using the Kaplan-Meier curve. In addition, the risk score model and clinical data (age, gender and tumor stage) were integrated to visualize the prognostic value of different patient characteristics. A nomogram was built to determine the accuracy and specificity of the model. In the final step, a similar method was applied for validation in the independent dataset (TCGA).

### Clinical verification of prognosis-related immune cells

#### Case collection

This study collected the clinical data of 116 CRC patients with initial treatment who received surgery from January 2016 to December 2020 and their postoperative tumor tissue paraffin-embedded samples. The data and specimens were obtained from the Department of Anorectal Surgery, Dongfang Hospital Affiliated to Shanghai Tongji University. Inclusion criteria: (1) patients without other malignant tumors before operation; (2) patients with CRC confirmed by postoperative pathology and with a pathological staging of I ~ IV; (3) patients who underwent radical tumor resection without anastomotic leakage, cardiovascular and cerebrovascular events or other complications; (4) patients with complete and standardized postoperative pathological reports and follow-up data. The survival time was defined as the time from the day when the patient was confirmed with CRC by the first biopsy or operation to the last follow-up or death of the patient. Of the 116 CRC patients, there were 71 males and 45 females, with an average age of 70 years old (33 ~ 93 years).

#### Immunohistochemical staining and counting

The tissue blocks sampled from 116 cases of CRC were fixed with 4% formaldehyde, conventionally dehydrated before embedding in paraffin and sliced into 4-µm sections Immunohistochemical of CD163, CD4 and CXCR5 were performed using the EnVision two-step method on the Dako Omnis automated immunohistochemical stainer. All target antigens were prepared with high-pressure sodium citrate buffer at pH 6.0. PBS was used in place of the primary antibody for a blank control, and already-known positive cases were used as positive controls. The specific steps were carried out according to the kit manufacturers instructions. Rabbit anti-CD4 antibody, mouse anti-CD163 antibody and mouse anti-CXCR5 antibody were provided by Abcam. Immunohistochemical sections were scanned by the pathological section scanner (3DHISTECH). Five 400-fold visual fields were randomly selected, and positive cells were counted with Image pro plus 6.0 software to measure the average value.

#### Statistical analysis

The χ^2^ test, Lasso regression and logistic regression analysis were adopted to screen variables, and rms package was used to generate a prediction model of a prognostic nomogram for risk assessment of CRC. All statistical analyses were performed using R language software and SPSS software (SPSS version 22). There was a statistical difference at P < 0.05 for all tests.

## Results

### Screening outcomes of prognosis-related immune cells based on GEO and TCGA databases

**Fig. 1:**
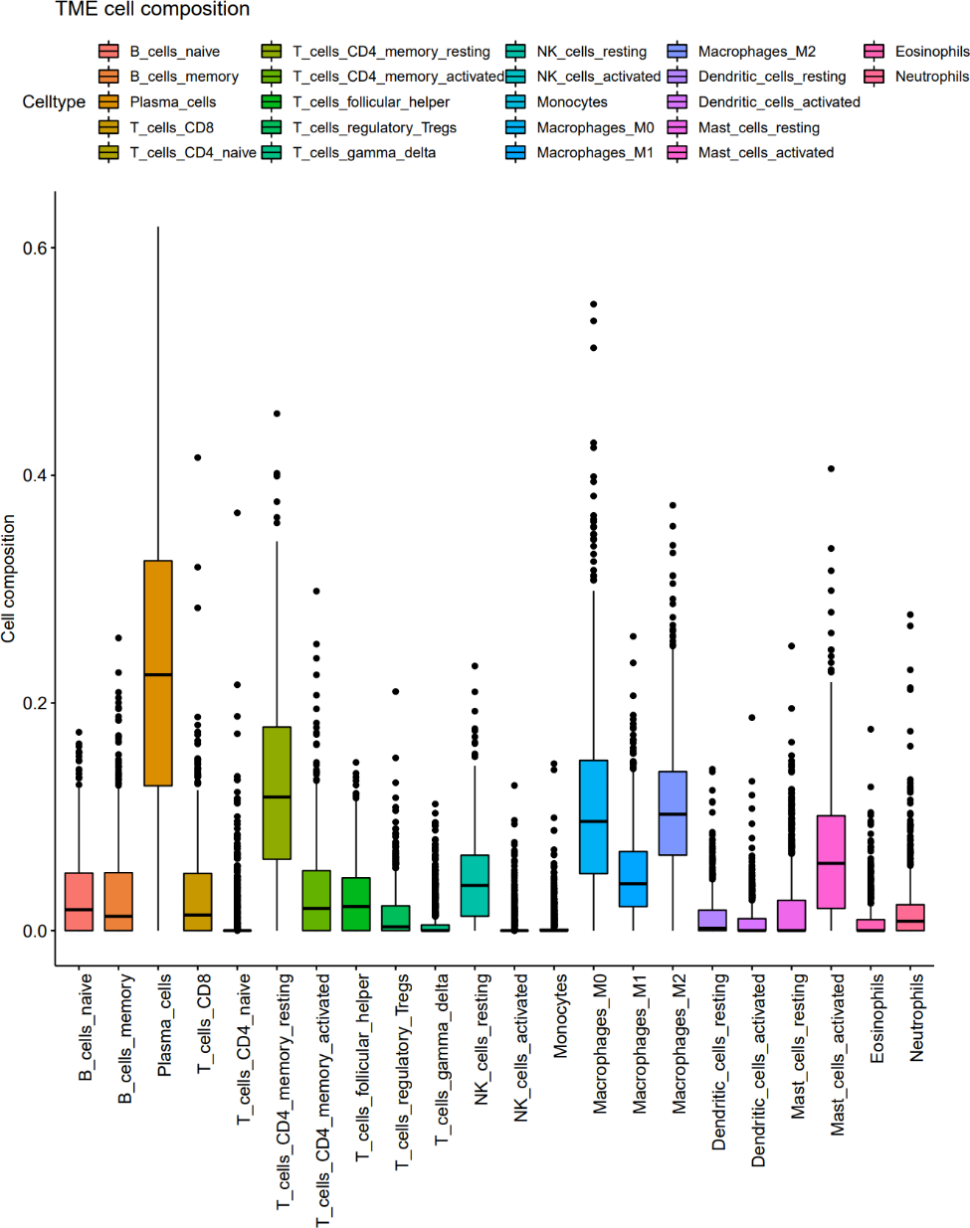
Analysis of the composition of 22 immune cells using CIBERSORT

2.1.1 Analysis of the composition of 22 immune cells using CIBERSORT: The mas5 was applied to homogenize the CELL data of GSE39582 and GSE41258 respectively, after which the two dataset matrices were merged and the batch effect was eliminated by using the sva R package, as shown in Fig. 1.

### Establishment of immune-related prognostic risk scoring model for CRC

**Fig. 2:**
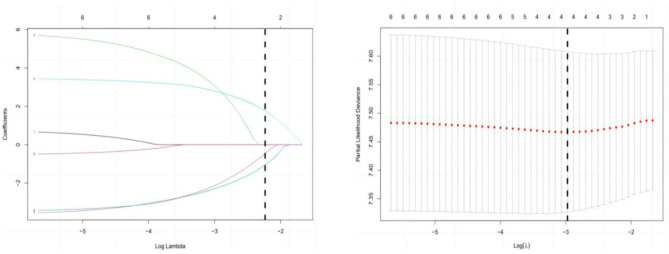
Removal of risk genes highly correlated with each other by using LASSO regression to enhance the reliability of the results and screen key prognosis-related immune cells based on the training set

**Fig. 3:**
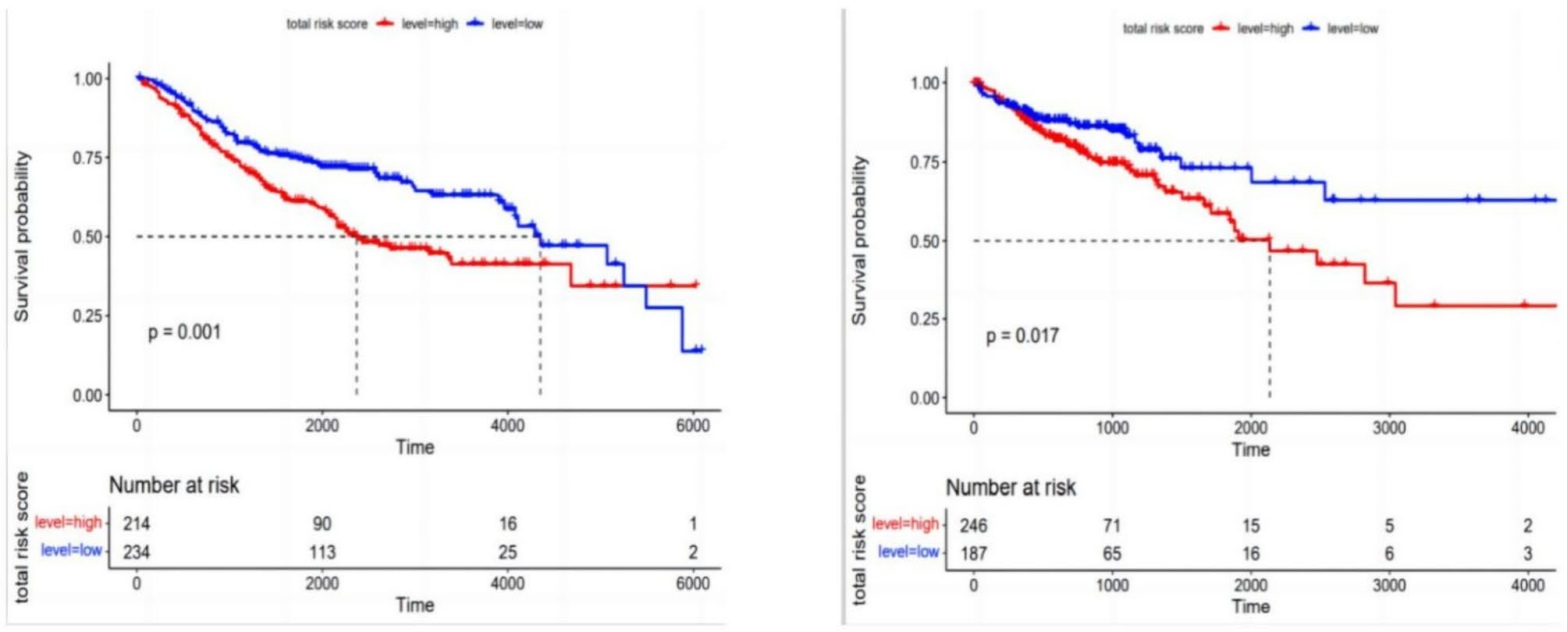
Kaplan-Meier curve analysis with the discovery of a significant difference in survival time between the high-risk group and low-risk group in the training set (left) and validation set (right)

Cell composition data of 456 samples in the training set were subject to univariate Cox regression analysis. The 6 immune cells related to OS were Naive B cells, Follicular Helper T cells (Tfh), Monocytes, M1 Macrophages, M2 Macrophages and Activated Mast Cells (all P < 0.05, Table 1). LASSO regression analysis was then performed to select the most suitable variables, with log (lambda. min) of -2.974318, and the number of the minimal-optimal variables [log (lambda. min)] of 4 (Fig. 2). Furthermore, the screened 6 prognosis-related immune cells were included in the multivariate Cox regression analysis, and 3 OS-related cells were finally selected as prognosis-related immune cells, which were Tfh cells, Monocytes and M2 Macrophages (Table 2). In the Cox model, the calculation of risk score followed the formula of (risk score) = Th* (-8.66597) + Monocytes*10.50211 + M2 Macrophages *3.174575. After that, the prognostic risk scores of each patient in the training set and validation set were calculated respectively, with the establishment of the low-risk group (234 cases in the training set, 187 cases in the validation set) and the high-risk group (214 cases in the training set, 246 cases in the validation set). In the training set, the 2,000-, 4,000- and 6,000-day survival rates were 48.3%, 10.7% and 0.9% in the low-risk group, and 42.1%, 7.5% and 0.5% in the high-risk group, respectively. In the validation set, the 2,000-, 4,000- and 6,000-day survival rates were 34.8%, 8.6% and 1.6% in the low-risk group, and 28.9%, 6.1% and 0.8% in the high-risk group, respectively. Survival curve analysis revealed that the prognosis of patients in the high-risk group was worse than that in the low-risk group (P < 0.05, Fig. 3).

**Table 1:** Univariate Cox regression analysis of 6 immune-related genes related to the overall survival of CRC

	HR	HR95L	HR95H	p.value
Naive B cells	0.003582	4.69E-05	0.273378081	0.010886
Follicular Helper T cells	3.14E-05	5.19E-08	0.018970756	0.001507
Monocytes	149198.4	97.14646	229140267.7	0.00146
M1 Macrophages	0.003361	4.48E-05	0.252394992	0.009745
M2 Macrophages	17.00275	1.136849	254.2935066	0.040083
Activated Mast Cells	20.76317	1.589435	271.2340997	0.020702

**Table 2:** Multivariate Cox Analysis of 4 risk genes in the prognosis risk scoring model of CRC

	coef	exp(coef)	lower 0.95	upper 0.95	p.value
Follicular Helper T cells	-8.66597	0.000172	1.35E-07	0.219578	0.017523
Monocytes	10.50211	36392.28	13.02138	1.02E + 08	0.00949
M1 Macrophages	-3.72874	0.024023	0.0002	2.879651	0.126795
M2 Macrophages	3.174575	23.91666	1.692086	338.0483	0.018815

#### Validation of immune-related prognostic risk scoring model for CRC

**Fig. 4:**
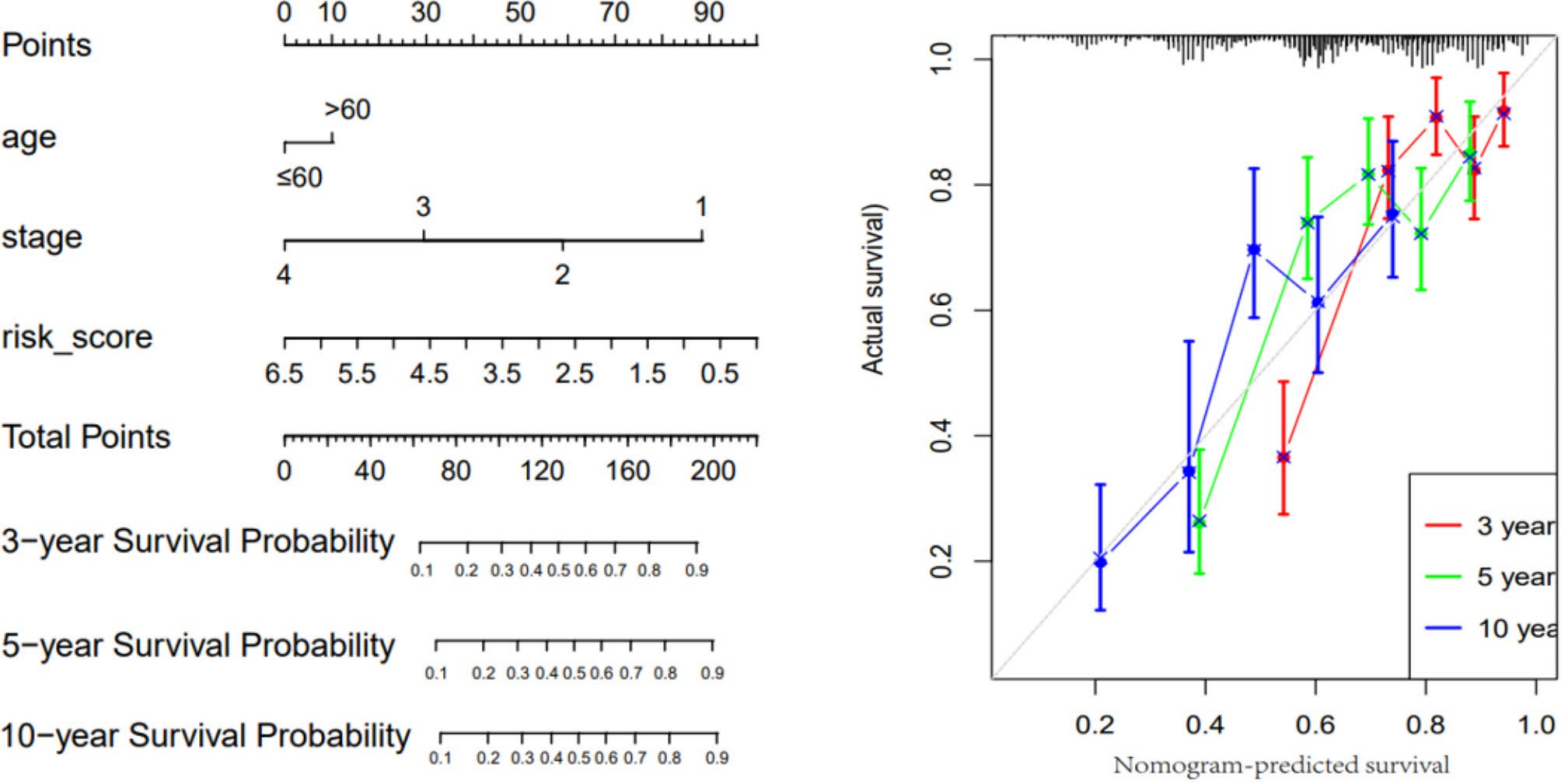
Comparison of the 3-year, 5-year and 10-year survival probabilities via calibration chart, showing that the actual survival rate was close to the predicted survival rate

**Fig. 5:**
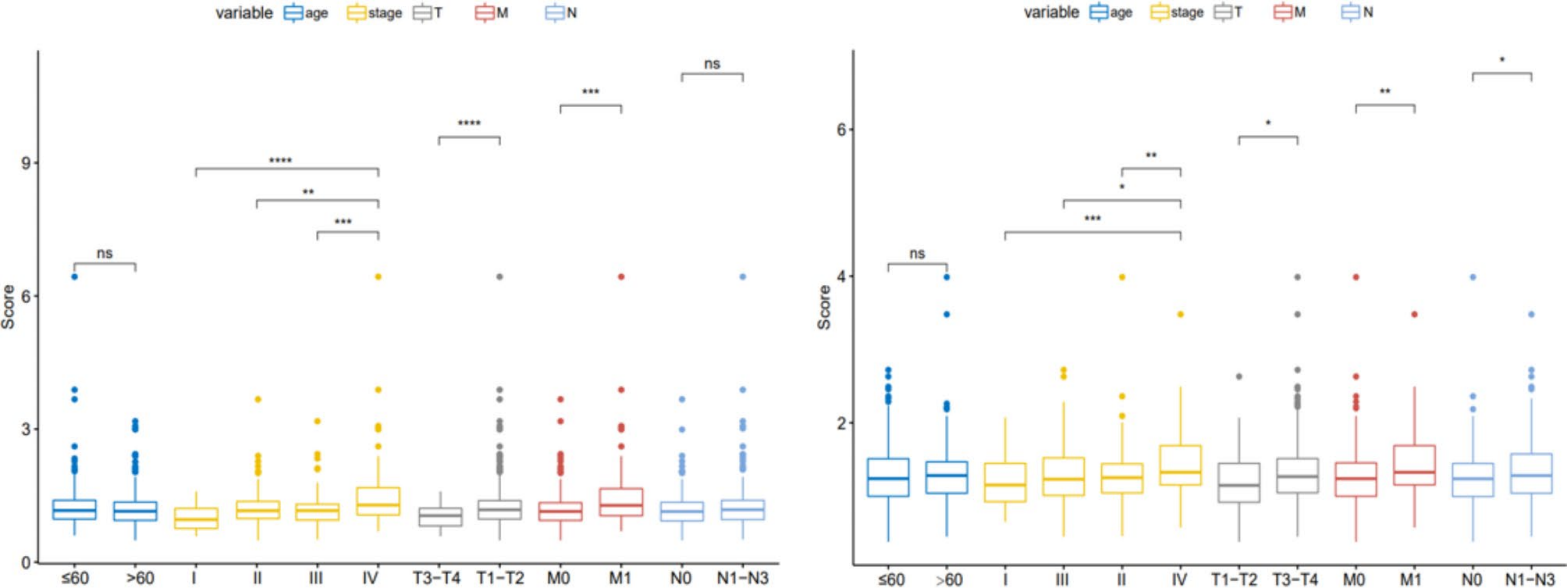
Comparison of differences between clinical data and risk scores in the training set and validation set

In the construction of the nomogram, the patient’s age, TNM stage and risk score were taken as scoring items. The final total score could be used to predict the 3-, 5- and 10-year OS of CRC patients. Compared with the TNM stage alone, the combined risk score indicated significant prognostic value (Fig. 4), suggesting that risk scores can be used to enhance the validity of the traditional TNM stage in predicting the prognosis of CRC. Furthermore, the GSE training set and TCGA validation set were grouped according to the median risk score of 1.182838 to compare the correlation differences of age, stage, and TNM stage with different risk score groups. It was found that the risk score correlated primarily with stage (Fig. 5).

### Clinical validation of prognosis-related immune cells

#### The relationship of CD163 expression with the prognosis and clinicopathological parameters of CRC patients

**Fig. 6:**
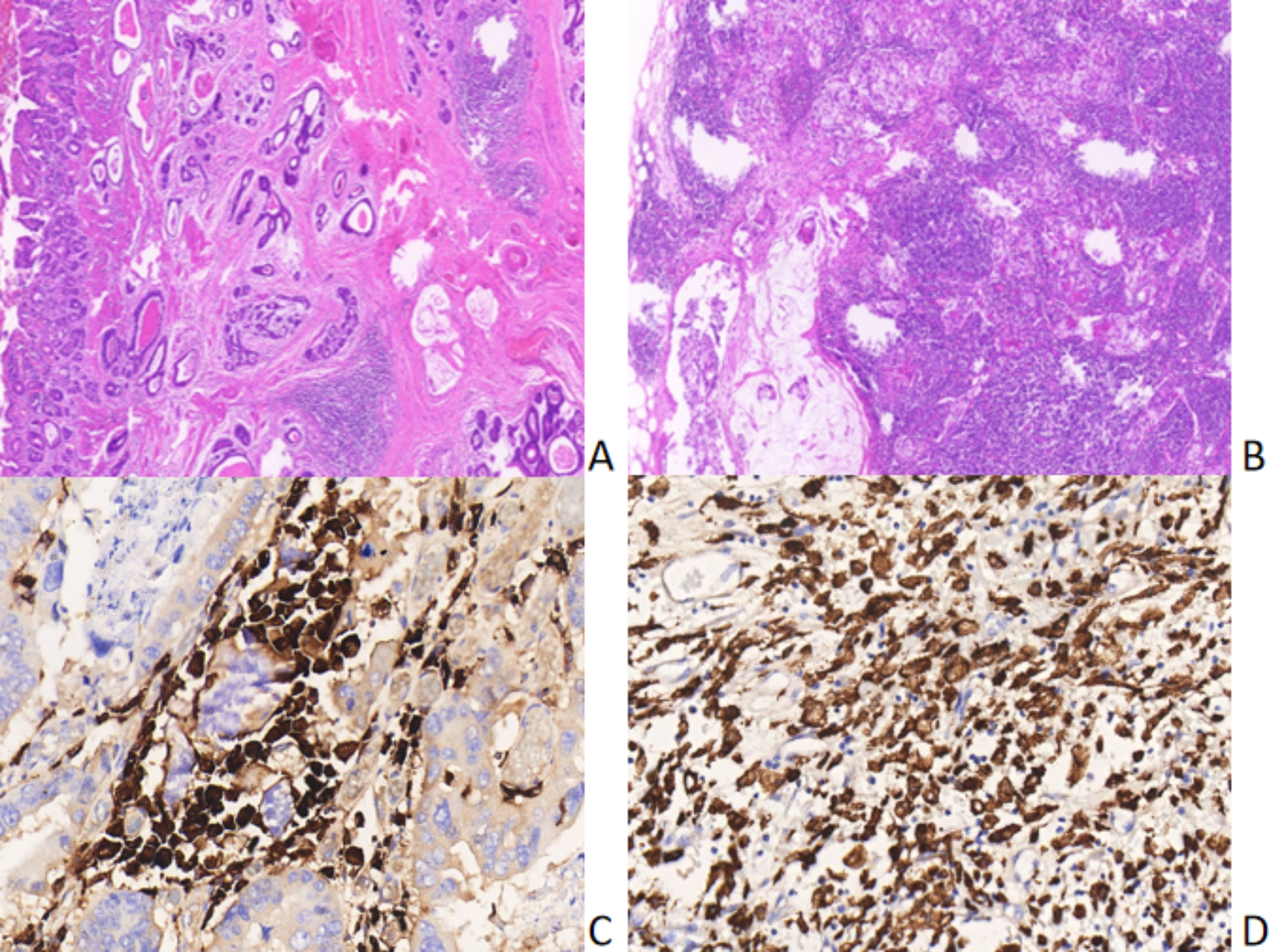
Lymphocyte infiltration and CD163 protein expression in rectal cancer A Pericancerous focal lymphocyte infiltration; B Diffuse lymphocytic infiltration in the stroma of cancer tissue; C CD163 protein expression in the stroma of cancer tissue; D CD163 protein expression in the stroma of paracancerous tissue

**Fig. 7:**
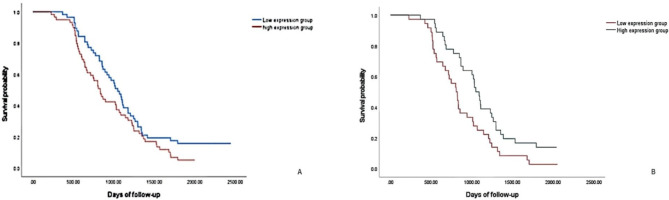
A Comparison of the overall survival curve between patients with high and low expression of CD163 protein in CRC

CD163 marker was located in the cytoplasms of Monocytes and M2 Macrophages cells, displaying as brown colored granules.(Fig. 6), and could be slightly expressed in normal mucosa, paracancerous tissue and adenoma tissue. CD163-positive cell count in CRC was significantly different from that in normal mucosa, adjacent tissues and adenoma tissues, with statistically significant differences (130.01 ± 45.24 vs. 40.64 ± 15.28, 70.25 ± 33.04, and 85.21 ± 44.69, P < 0.05). Two groups of high-expression and low-expression were established with the median of CD163 protein expression as the cut-off value (118 cells/40HPF). The 1,000- and 2,000-day survival rates were 56.1% and 7.0% in the CD163 low-expression group, and 40.7% and 1.7% in the high-expression group, respectively, showing a worse prognosis in the high-expression group than that in the low-expression group (P < 0.05, Fig. 7A). These results suggest that the counts of Monocytes and M2 Macrophages may be negatively correlated with patient prognosis, indicating that CD163 can be a prognostic predictor of CRC. In addition, there was no direct relationship of CD163 protein expression with patients’ age, gender, tumor location, tumor diameter, degree of differentiation, TNM stage, presence/absence of signet-ring cells, and presence/absence of neural and vascular invasion (all P > 0.05, Table 3).

B Comparison of overall survival curve between patients with high and low expression of CD4 + CXCR5 protein in CRC.

**Table 3:** The relationship between CD163 expression and clinicopathological parameters in CRC

	Low expression group N=57	High expression group N=59	P value
Age (year), x ± s	70 ± 10.5	71 ± 10.4	0.63
**Gender n (%)**			
Male	33	38	0.41
Female	24	21	
**Tumor location n (%)**			
left	41	37	0.27
right	16	22	
**Differentiation degree n (%)**			0.29
High	2	2	
Moderate	52	50	
Poor	3	7	
**TNM stage n (%)**			0.35
I-II	15	20	
III-IV	42	39	
**Tumor diameter n (%)**			0.93
≥ 5 cm	28	30	
≥5 cm	29	29	
**Lymphatic metastasis n (%)**			0.49
No	37	23	
Yes	21	36	
**Signet-ring cell**			0.39
Yes	4	2	
No	53	57	
**Nerve/vascular invasion**			0.09
Yes	20	30	
No	37	29	
**Microsatellite**			0.33
stability	51	55	
instability	6	4	

#### The relationship of CD4 + CXCR5 expression with the prognosis and clinicopathological parameters of CRC patients

CD4 + CXCR5 marker was located in the nucleus of Tfh cells, displaying as brown colored granules. Similarly, high-expression and low-expression groups were constructed based on the cut-off value of the median of CD4 + CXCR5 protein expression (212 cells/40HPF). The 1,000- and 2,000-day survival rates were 63.9% and 5.6% in the CD4 + CXCR5 high-expression group, and 33.3% and 2.8% in the low-expression group, respectively, with a better prognosis in the high-expression group than that in the low-expression group (P < 0.05, Fig. 7B). These data suggest that Tfh cell count may be positively related to the prognosis of patients, and CD4 + CXCR5 can be used as a prognostic predictor of CRC. Besides, no direct relationship of CD4 + CXCR5 protein expression with patients’ age, gender, tumor location, tumor diameter, degree of differentiation, TNM stage, presence/absence of signet-ring cells, and presence/absence of neural and vascular invasion (all P > 0.05, Table 4).

**Table 4:** The relationship between CD4 + CXCR5 expression and clinicopathological parameters in CRC

	Low expression group N=57	High expression group N=59	P value
Age (year), x ± s	69.7 ± 10.6	64.8 ± 13.5	
**Gender n (%)**			0.23
Male	32	40	
Female	26	18	
**Tumor location n (%)**			0.62
left	42	39	
right	16	19	
**Differentiation degree n (%)**			0.37
High	3	3	
Moderate	44	47	
Poor	11	8	
**TNM stage n (%)**			0.19
I-II	19	11	
III-IV	39	47	
**Tumor diameter n (%)**			0.48
≥ 5 cm	23	27	
≥5 cm	35	31	
**Lymphatic metastasis n (%)**			0.62
No	21	18	
Yes	37	40	
**Signet-ring cell**			0.17
Yes	2	6	
No	56	42	
**Nerve/vascular invasion**			0.16
Yes	29	21	
No	29	37	
**Microsatellite**			0.56
stability	56	55	
instability	2	3	

## Discussion

The term tumor microenvironment (TME) was first proposed by Ioannides and Whiteside, which, by definition, is the local biological environment in the process of tumor development and progression [9]. Tumor cells and the TME are functionally indispensable to each other. While the TME supports tumor cell growth and development by modulating its architecture and presenting barriers that contribute to immune privilege, tumor cells are capable of changing the TME. The TME, especially its role in tumorigenesis and progression, has been of increasing interest in the field of oncology. Collectively, the TME facilitates the growth of tumor cells by providing structural support, promoting the development of anti-tumor drug resistance, and assisting tumor cells in escaping immune surveillance via local immune response suppression [10].

Macrophages have been reported to be the most popular immune cell subset in the research of TME. At the site of tumor or inflammation, differentiated Macrophages can polarize into various subtypes as the environment changes. Macrophage polarization is a dynamic process, which depends on changes in local microenvironment and can be regulated by various intracellular signaling molecules and pathways. On the contrary, with the change in the phenotype of Macrophages, the genes expressed by Macrophages and their secreted cytokines will also change accordingly, thus affecting the local microenvironment [11]. In view of corresponding phenotype and function, Macrophages can be divided into classically activated Macrophages (M1) and alternatively activated Macrophages (M2).While M1 macrophages stimulate tumor antigen presentation, M2 macrophages inhibit tumor-antagonizing immunocyte‐killing activity via interleukin‐1β (IL‐1β), interleukin‐6 (IL‐6), and transforming growth factor‐β (TGF‐β) production [12, 13].In addition, there are three additional subtypes of Macrophages, including tumor-associated Macrophages (TAM), CD169 + Macrophages and TCR + Macrophages. Monocytes are derived from common myeloid precursors (CMPs). Under the action of chemokines secreted by stromal cells and tumor cells in TME, Monocytes are recruited to the tumor site and further differentiate into TAM. According to multiple studies, TAM plays an important role in tumor growth, invasion and metastasis, and its degree of infiltration exhibits an intimate association with the prognosis of patients [14–16]. The phenotype of TAM is adjustable in tumor progression, and it is similar to the M1 phenotype at the initial stage. At the advanced stage, TAM can be changed into an M2 phenotype by recruiting Monocytes via secreting chemokines (e.g., CCL2, CCL5, CCL7, CXCL8 and CXCL12), and polarizing under the stimulation of IL4, IL6, IL10, IL13 and TGFβ[15–17]. As a cell surface marker, CD163 is highly expressed in M2-type Macrophages, which is considered to have the ability to distinguish the M2 phenotype from other M1 phenotypes [18, 19]. In addition, TAM and M2 Macrophages can promote chronic inflammation, angiogenesis, and tumor growth by secreting various cytokines, growth factors (e.g., EGF, VEGF, PDGF, FGF and TGFβ), matrix metalloproteinases (MMPs), M-CSF, etc. [20–24].

Tumor-infiltrating lymphocytes (TILs) are important immunocytes found in tumor tissue. As the major component of tumor‐infiltrating immunocytes, TILs consist of T (CD3^+^), B (CD19^+^), and natural killer (NK, CD16^+^, and CD56^+^) cells [25]. T cells can be subsetted into four types by their functions and surface markers: cytotoxic (Tc), helper (Th), regulatory/suppressor (Treg/Ts), and memory (Tm) T cells. CD4^+^ and CD8^+^ T cells are two predominant T cell subsets in the TIL family, including CD8^+^ Tc cells, CD4^+^ Th cells, Foxp3^+^ Treg cells, CD45RO Tm cells, and natural killer T cells [26]. CD8^+^ Tc cells either kill tumor cells directly or promote the progression of inflammatory response via interleukin‐17 production [27].Naive CD4 + T cells are activated by antigens presented by dendritic cells and differentiate into different effector T cells under the action of different transcription factors and cytokines. In the case of expressing chemokine receptor (CXCR) 5, T cells will migrate to the periphery of follicular B cells and further differentiate into Tfh cells; while if T cells receive signals from Th1, Th2 or Th17 cells, CD4 + T cells will differentiate into Th1, Th2 or Th17 cells [28–30]. Among them, Tfh cells have been identified as a new T helper subset specialized to promote the differentiation and maturation of B cells, induce germinal centers, and promote the maturation of plasma cells and memory B cells [31–33]. Nevertheless, it is still unclear with respect to the role and pathogenesis of Tfh cells in tumors. According to prior research, naive T cells do not express CXCR5, only activated T cells can express CXCR5 temporarily, and Tfh cells are the only type of T cells that can consistently overexpress CXCR5, suggesting that CXCR5 is an important surface marker of Tfh cells [30, 34–36]. CD4^+^Treg cells that are characterized by Foxp3 expression are capable of inhibiting Te cells. CD4^+^, CD25^+^, and Foxp3^+^ Treg cells are responsible for the immunosuppressive mechanisms by which the body maintains immune tolerance and homeostasis [37]. Tm cells in a resting state can be identified by anti‐CD45RO Tm cells. Low expression levels of activated surface markers such as CD25, major histocompatibility complex class II antigen, CD54, and CD26 are typically found in CD45RO Tm cells, which suggests that the Tm cells may be newly activated. In this context, low‐dose persistent or cross-antigen exposure presumably stimulates CD45RO Tm cells constantly and thereby leads to their long‐term survival [38]. In short, these immune molecules contribute to tumorigenesis and progression via numerous pathways.

TILs are associated with cancer prognosis and the efficacy of anticancer therapy [39, 40]. Moreover, a growing body of research has investigated TILs as prognostic or diagnostic markers of breast cancer and discovered that in TILs are ubiquitous in TNBC and HER2^+^ breast cancer cells, which indicates a favorable prognosis [41, 42]. Additionally, TILs are proven markers of response to neoadjuvant chemotherapy. However, the prognostic value of TILs varies by the molecular subtypes of breast cancer. In the presence of TILs, patients with intestinal cancer are reported to have improved survival [43–45]. In contrast, there is literature arguing that TILs are not predictive of survival in patients with esophagus and/or colorectal cancer [46]. While tumor-infiltrating immunocytes are known to play a role in cancer progression and invasion, their value as a prognostic factor for survival remains controversial [43, 47]. The oral squamous cell carcinoma study by Shaban et al. reported TILs as a prognostic indicator for disease‐free survival [48]. In another study, TILs were found to have prognostic significance in melanoma [49]. Likewise, TILs are reportedly prognostic for esophagus cancer and bile duct cancer [50, 51]. Furthermore, TILs can potentially guide the treatment of breast cancer as prognostic factors and markers of breast cancer response to chemotherapy [52]. All these findings provide evidence for the potentially crucial effects of tumor‐infiltrating immunocytes on cancer prognosis. However, different types and levels of prognostic immunocytes might be brought into play to cope with highly heterogeneous tumors in various body parts and stages. This reveals the need for further research to guide clinical cancer treatment with ideal prognostic models.

In conclusion, our study suggests that Monocytes and M2-type Macrophages correlate negatively, while Tfh cells correlate positively with the prognosis of CRC patients through bioinformatic analysis and clinical validation, although there is still a poor understanding of the mechanism of immune cells in TME so far. Both CD163 and CD4 + CXCR5 can be regarded as prognostic predictors for CRC patients to facilitate the evaluation of cancer prognosis and TME status, thereby optimizing individualized treatment plans and improving cancer patient survival.

## Data Availability

The datasets generated and analysed during the current study are downloaded from TCGA (https://portal.gdc.cancer.gov) or GEO (https://www.ncbi.nlm.nih.gov/geo).
